# Revisiting the physical mutagenesis for sugarcane improvement: a stomatal prospective

**DOI:** 10.1038/s41598-020-73087-z

**Published:** 2020-09-29

**Authors:** Shafquat Yasmeen, Muhammad Tahir Khan, Imtiaz Ahmed Khan

**Affiliations:** Sugarcane Biotechnology Group, Nuclear Institute of Agriculture (NIA), Tandojam, 70060 Pakistan

**Keywords:** Genetics, Plant sciences

## Abstract

This study was conducted to investigate the influence of gamma rays on stomatal parameters and the interaction of these traits with agronomy of the sugarcane crop. Three genotypes of sugarcane (NIA-0819, NIA-98, and BL4) were exposed to four doses of gamma radiation (10, 20, 30, and 40 Gy) and then subjected to field trials. Stomatal length, width, and density were observed using scanning electron microscopy. Moreover, agronomic and sugar-related characteristics of the crop were determined at maturity. The stomatal parameters augmented at two lower doses of gamma radiation (10 and 20 Gy) and declined under higher doses (30 and 40 Gy). The maximum stomatal length was observed in NIA-0819 at 10 Gy (63.71 µm), whereas NIA-98 and BL4 demonstrated highest stomatal length under 20 Gy (54.11 and 57.40 µm, respectively), indicating a role of genetic factors in varietal response. Similar trend was noticed for stomatal width and density as well. The lowermost treatment (10 Gy) of NIA-0819 produced maximum stomatal density (115.31 stomata mm^-2^ on the abaxial surface). Adaxial stomatal density was significantly lower than the abaxial one. Sugar quality attributes revealed a different tendency. Sucrose contents of BL4 increased gradually from 12.33% at control to 14.54% at 40 Gy. Yield and yield-contributing traits of genotypes indicated a strong positive correlation with the stomatal parameters. The present study suggested that gamma radiations cause variations in stomatal characteristics of sugarcane. These changes further influence the photosynthetic activity and instigate a direct impact on the agronomic performance of the crop.

## Introduction

Over the course of evolution, plants have adopted numerous ways of stress tolerance. Such mechanisms include the modulation of the life cycle, changes in CO_2_ assimilation and water transpiration, and modifications in growth and development. Many of these responses relate to the imperative role played by stomata^1^. These specialized plant structures, bordered by parenchymatic guard cells, are responsible for gaseous exchange and water transpiration. Efficacy of stomata depends on its size, shape and number, and is reflected in terms of photosynthesis. Hence, stomatal density and size, both are relevant factors towards ultimate crop yields^2^.

Water deficiency significantly reduces the yields of commercial crops. Such a decline in yields is more evident in the case of water demanding crops, for instance sugarcane, rice, cotton, and maize^3,4^. Under such conditions, sugarcane manifests variations in transpiration rate, stomatal conductance, internal CO_2_ concentration, and photosynthetic rate—primarily instigated through stomata^5^. Stomatal openings, therefore, play an important part in coping with adverse environmental circumstances, maintaining growth and development, and regulating the rate of transpiration. Stomatal initiation and development are mainly controlled by genetic factors. Therefore, the mutagenesis in the DNA of the plants can lead to changes in stomatal parameters^6^. The mutation breeding has significantly contributed to crop improvement^7^. Various crop varieties have been released around the world for commercial cultivation using this approach^8,9^. Mutation breeding has served in increasing crop yields and economic returns to the farmers, and availability of diversified genetic stock to breeders^10^. For mutagenesis of crop plants, different mutagenic agents can be used such as gamma rays, X-rays, and chemical agents^11^. The mutagenic changes cause variations in plant growth, height, leaf characteristics, and several biological and physiological traits including stomatal size and number^12^.

Any change in stomatal structure, the major cellular component of plants towards growth and development and stress tolerance, is of significant importance. Therefore, exploring the vicissitudes in stomata as a result of physical mutagenesis can provide interesting insights into its functionality. In such investigations, scanning electron microscopy (SEM) is an excellent technique to explore the anatomy of plant tissues. It can help in studying many of the plant structures including stomata, papillae, and trichomes, etc. and variations induced in these plant structures can be thoroughly examined.

Sugarcane is a tropical crop with C4 photosynthetic metabolism. It is a huge biomass producer attributed to its efficient photosynthetic system. Lakshmanan and Robinson^13^ illustrated that sugarcane is extremely sensitive to water deficit conditions. Moreover, Bertolino^14^ also described that sugarcane productivity is highly water-dependent. The water limiting environment can reduce the cane yield by up to 60%^4,15^. In the recent past, sugarcane has gained popularity towards several new industries causing a surge in its production in the world. Previously, its cultivation was intended for sugar production only. However, currently, ethanol, press mud, and bagasse are also being produced^16,17^. Sugarcane’s role has also been well recognized towards bioenergy engenderment^18–20^. Nevertheless, due to intensive cropping and climate change, water scarcity has become one of the most severe issues in agriculture, and sugarcane is not an exception to it. The situation, therefore, demands the evolution of resilient varieties that could sustain such stress and produce economic yields.

The present study was initiated to investigate the structural changes in stomata caused by physical mutagenesis. It has been reported earlier that gamma radiations alter stomatal characteristics of certain plants^21–23^. Since stomatal parameters are associated with plants’ agronomic performance^24–27^ and stress tolerance^28–30^, we hypothesized that changes in stomatal traits, induced through gamma radiations, would correlate to agronomic performance of the sugarcane crop. In this study, three genotypes of sugarcane, having different timelines for maturity, were exposed to four doses of gamma radiation and then subjected to field trials for 2 years. Yield and yield contributing traits, as well as sugar yield and its components, were studied and compared against the changes in stomatal aperture and density through electron microscopy. As per our knowledge, this is the first report investigating the direct impact of mutagenesis on sugarcane stomata through SEM. Therefore, the study carries immense importance in sugarcane breeding and stress tolerance research.

## Material and methods

The study comprised of three major components; mutation induction, field evaluation, and stomatal characteristics analysis. Three genotypes of sugarcane viz. NIA-0819 (early maturing), BL4 (mid maturing), and NIA-98 (late maturing) were subjected to gamma irradiation. The double budded sets of each genotype were radiated employing four doses of gamma rays (10, 20, 30, and 40 Gy) at a rate of 30.86 Gy min^−1^. The doses were selected on the basis of LD_50_ of sugarcane, which has been proposed to be 30–50 Gy^31^. Prior studies have also reported inducing influence of lower doses of gamma radiation and damaging effects around LD_50_^32,33^. The Co^60^ radiation source (GB100-80, Canada) of the Nuclear Institute of Medical and Radiotherapy (NIMRA), Jamshoro was used for radiating the cane sets. Subsequently, the mutants were subjected to field evaluation using the experimental layout of randomized complete block (RCB) design with four replications. The irradiated material was planted at the experimental farm of Nuclear Institute of Agriculture (NIA), Tandojam, Pakistan along with the control (non-irradiated). The trials were conducted maintaining a plot size of 25 × 5 m and 1.5 m row to row distance. The crop was sown in the month of September each year (cropping season 2016–17 and 2017–18). NPK fertilizers were applied at a recommended rate of 200, 120, and 150 kg ha^−1^, respectively^34^. Recommended agronomic practices (weeding, fertilizer application, earthing up, and irrigation) were followed throughout the growth period^35^.

The agronomic parameters viz*.* plant height, number of stools plant^−1^, girth, stool weight, number of internodes, internode length, leaf width, and leaf length were recorded at the age of 12 months. The juice quality-related characteristics i.e. brix %, fiber %, sucrose %, commercial cane sugar % (CCS), and purity % were analyzed by randomly taking five stools from each plot. The sugar contents were analyzed according to the method reported earlier^36^. Brix % was estimated using Anchor, China Yuyao No. 2 brix hydrometer, while sucrose % was determined through Bellingham & Stanley ADP 220 Polarimeter (United Kingdom). Moreover, fiber % was investigated by analyzing the differences between fresh and dried fiber of the sugarcane using Ohaus, Explorer (Switzerland) semi-micro balance. Three rows from each plot were harvested to record yield data.

The electron microscopy was done using the Scanning Electron Microscope facility of the Central Research Laboratory, University of Karachi. Ten mature, two and half-month-old leaves were collected from each treatment for analysis. The leaves were mounted on metallic stub using adhesive tape, and then gold plated in the sputtering chamber for 6 min and studied under SEM. Two sections of the leaf, one from the lower surface and one from the upper surface were analyzed using SEM Jeol, JSM T-200, and Jeol-T6380 at a voltage of 5–15 kV with distinct magnification. For measuring the stomatal dimensions, multiple readings were taken from each sample. The reading of the ocular micrometer was calibrated using standard stage, and the stomatal length, width, and density were determined from permanent slides. The stomatal dimensions were also analyzed using the SEM measuring bar^37,38^.

The experimental data were subjected to the factorial arrangement of analysis of variance (ANOVA) under linear models of statistics to investigate the statistical differences among different traits by using computer program, Student Edition of Statistix (SWX), Version 8.1*.* Further, the least significant difference (LSD) test was also applied to test the level of significance among different combinations means^39^. Association of the gamma doses of radiation and sugarcane parameters was also explored. For this, coefficients of determination, as well as coefficient of correlation, were ascertained. Regression analysis of the mid-region against stomatal and agronomic characteristics of the crop was conducted using Microsoft Excel v. 2019. The coefficient of correlation and its p values were dissected employing the statistical package of SPSS v. 21 on Windows operated system.

## Results

**Figure 1:**
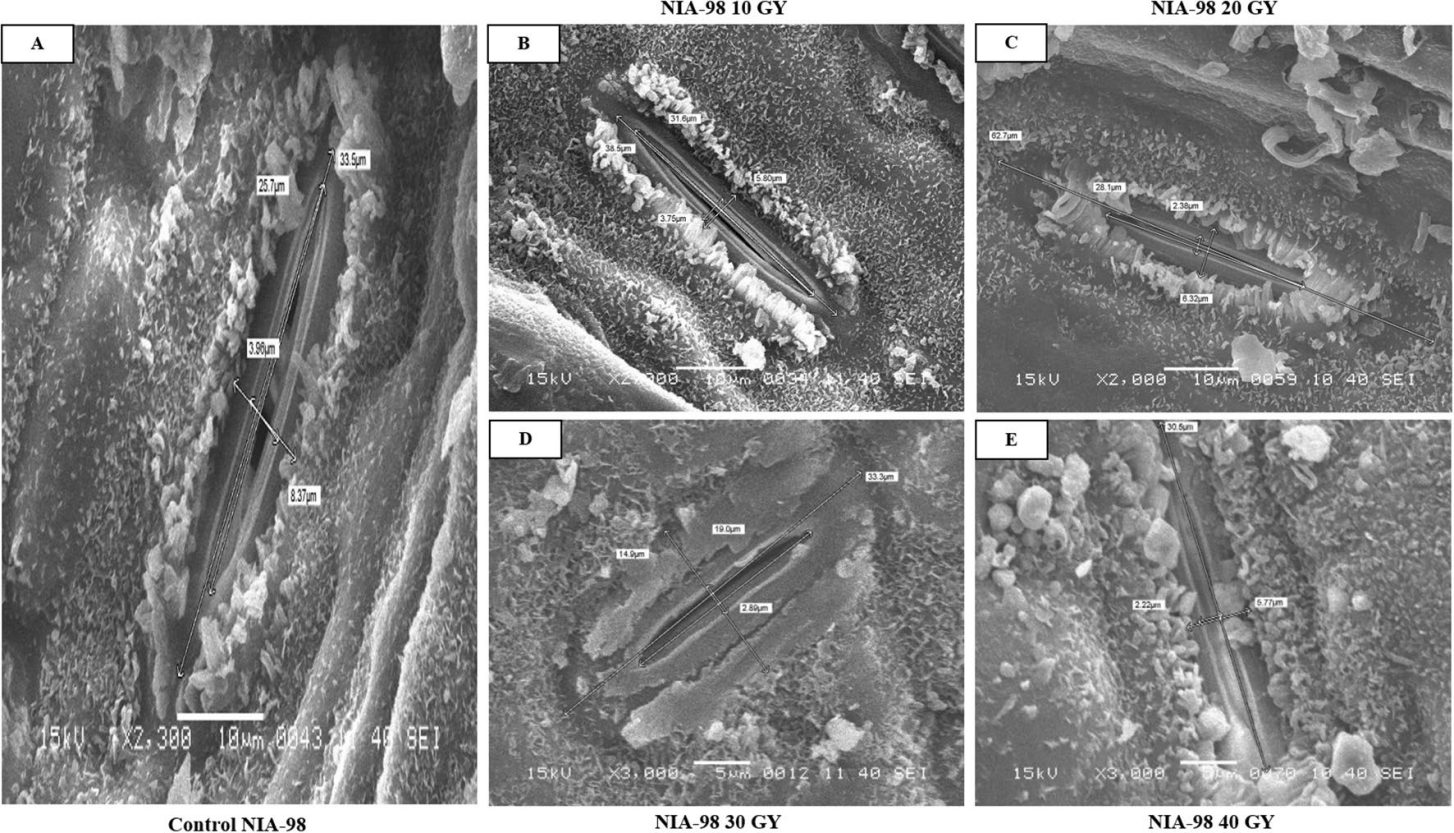
Effect of physical mutagenesis on stomatal dimensions of NIA-98: sugarcane variety NIA-98 was subjected to four different doses of gamma radiations viz. 10, 20, 30, and 40 Gy. (**A**) Stomata of NIA-98 under control; (**B**) stomata of NIA-98 under 10 GY; (**C**) stomata of NIA-98 under 20 GY; (**D**) stomata of NIA-98 under 30 GY; (**E**) stomata of NIA-98 under 40 GY. Scanning electron microscopy of the stomata of treated sugarcane plants revealed that gamma radiations led to changes in stomatal dimensions.

**Figure 2:**
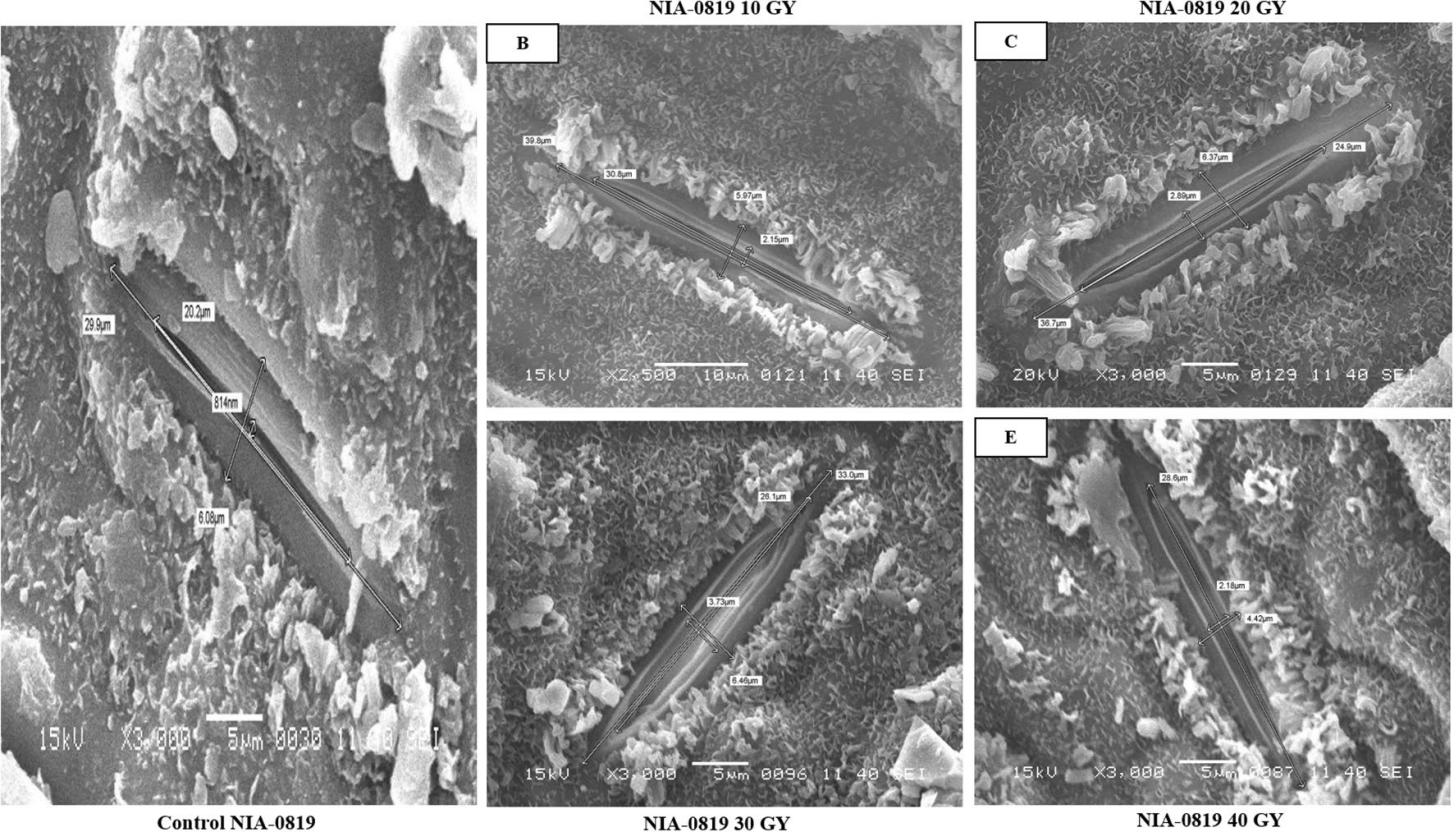
Effect of physical mutagenesis on stomatal dimensions of NIA-0819: sugarcane variety NIA-0819was subjected to four different doses of gamma radiations viz. 10, 20, 30, and 40 Gy. (**A**) Stomata of NIA-0819under control; (**B**) stomata of NIA-0819under 10 GY; (**C**) stomata of NIA-0819under 20 GY; (**D**) stomata of NIA-0819under 30 GY; (**E**) stomata of NIA-0819under 40 GY. Scanning electron microscopy of the stomata of treated sugarcane plants revealed tat gamma radiations led to changes in stomatal dimensions.

**Figure 3:**
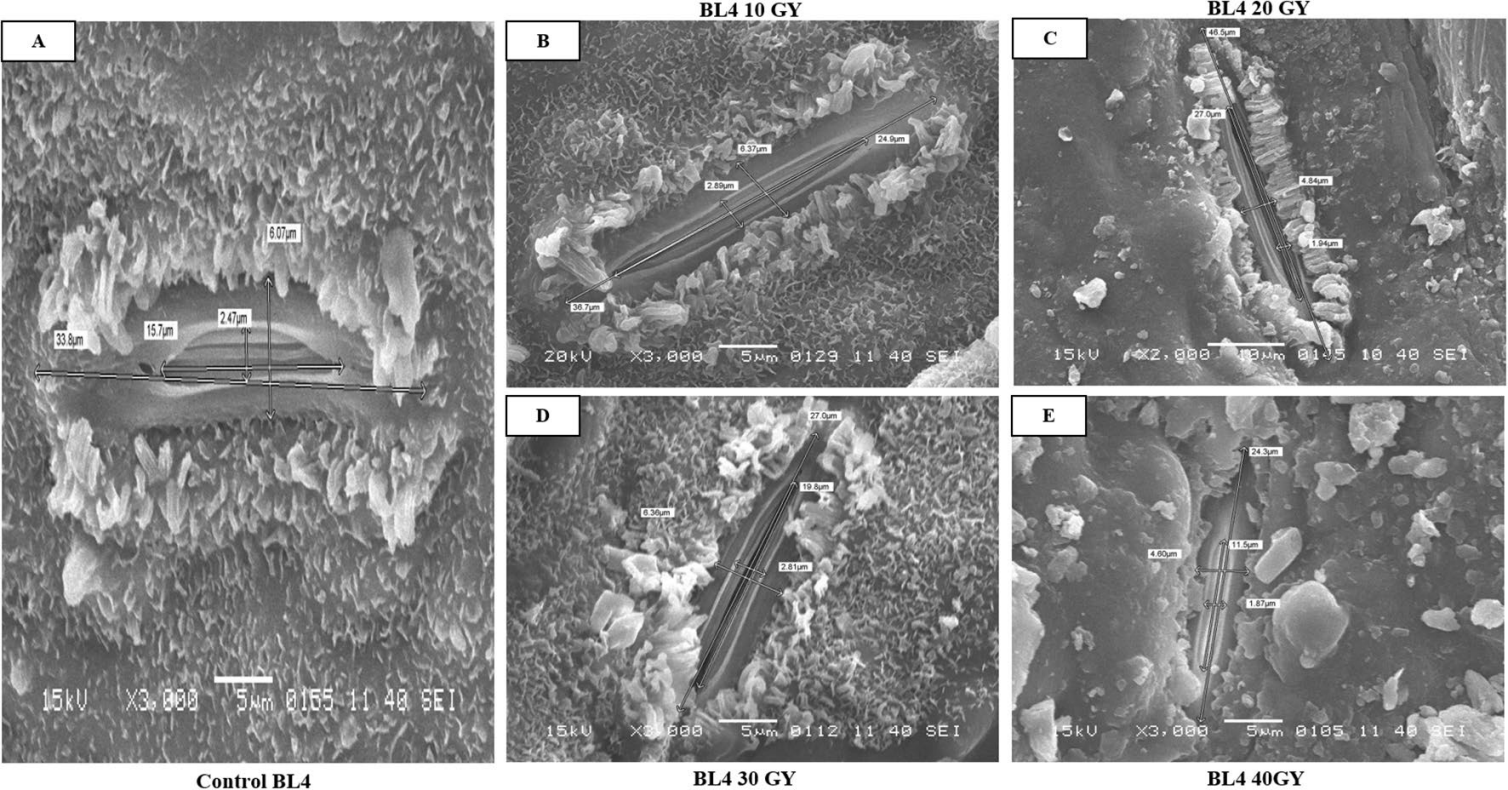
Effect of physical mutagenesis on stomatal dimensions of NIA-0819.Sugarcane variety BL4was subjected to four different doses of gamma radiations viz. 10, 20, 30, and 40 Gy. (**A**) Stomata of BL4under control; (**B**) stomata of BL4under 10 GY; (**C**) stomata of BL4under 20 GY; (**D**) stomata of BL4under 30 GY; (**E**) stomata of BL4under 40 GY. Scanning electron microscopy of the stomata of treated sugarcane plants revealed that gamma radiations led to changes in stomatal dimensions.

The data elucidated that the stomata of sugarcane leaves, when subjected to gamma radiation, exhibited morphological changes on both the adaxial and abaxial surfaces. Significant differences in the length and width of the stomatal aperture were observed in sugarcane plants whilst exposed to gamma radiation. On the adaxial surface, the maximum stomatal length was observed at 10 Gy (63.71 µm) in NIA-0819, and at 20 Gy in NIA-98 (54.11 µm) and BL4 (57.40 µm) (Table 1; Figs. 1, 2, 3). On the other hand, higher doses of gamma rays caused an extreme reduction in the stomatal length. The maximum diminution in stomatal aperture was observed in 40 Gy of NIA-98 (23.63 µm). NIA-0819 demonstrated the highest stomatal length in all doses on the adaxial surface as compared to other genotypes in the study. The average stomatal lengths of NIA-0819, BL4, and NIA-98 were 46.33, 38.47, and 42.47 µm, respectively.

**Table 1:** Mean values of stomatal length (µm) on the abaxial and abaxial leaf surface of the sugarcane genotypes.

Treatment	Stomatal length (µm)							
	Adaxial surface				Abaxial surface			
	NIA-0819	NIA-98	BL4	Means	NIA-0819	NIA-98	BL4	Means
Control	42.48 c	38.32 c	42.37 c	41.05	40.48 c	36.26 b	38.12 c	38.28
10 Gy	63.71 a	42.77 b	48.24 b	51.57	57.83 a	37.95 b	41.39 b	45.72
20 Gy	54.90 b	54.11 a	57.40 a	55.47	50.87 b	46.28 a	48.27 a	48.47
30 Gy	39.82 c	33.51 d	36.23 d	36.52	33.15 d	31.84 c	36.41 d	33.8
40 Gy	30.74 d	23.63 e	28.13 e	27.5	25.91 e	21.94 d	24.38 e	24.07
Means	46.333	38.471	42.475	–	45.933	34.857	37.717	–

Means followed by different letters in the same column indicate significant differences (p < 0.05).

The study revealed that the stomatal apertures on the abaxial surface were far shorter than those on the adaxial surface. The mean stomatal length was 45.93, 34.85, and 37.71 µm for NIA-0819, NIA-98, and BL4, respectively. Like adaxial stomatal aperture values, NIA-0819 showed higher stomatal lengths at 10 Gy (57.83 µm); BL4 (48.27 µm) and NIA-98 (46.28 µm) at 20 Gy. Contrarily, stomatal length for the abaxial surface at control was 40.48, 36.26, and 38.12 µm for NIA-0819, NIA-98, and BL4, respectively. The minimum value for stomatal length on the abaxial surface was observed in NIA-98 at 40 Gy (21.94 µm) (Table 1; Figs. 1, 2, 3).

The changes in stomatal width were also recorded through SEM. Generally, the stomatal width on both leaf surfaces decreased with the increasing exposure to gamma radiation. On the upper surface, NIA-98 showed the maximum adaxial stomatal width of 6.17 µm at 20 Gy (Table 2; Figs. 1, 2, 3). While, the adaxial stomatal width at the highest dose was 1.91, 2.90, and 3.34 µm for NIA-0819, NIA-98, and BL4, respectively. The abaxial stomatal width increased under low doses (10 and 20 Gy) and decreased at higher doses (30 and 40 Gy). The maximum abaxial stomatal width was recorded at 10 Gy in NIA-0819 (6.63 µm), whereas in case of NIA-98 and BL4, the highest values were observed at 20 Gy (7.63 and 6.35 µm, respectively).

**Table 2:** Mean values of the stomatal width (µm) on the adaxial and abaxial leaf surface of sugarcane genotypes.

Treatment	Width of stomatal aperture (µm)							
	Adaxial surface				Abaxial surface			
	NIA-0819	NIA-98	BL4	Mean	NIA-0819	NIA-98	BL4	Mean
Control	5.10 b	3.35 c	4.71 ab	4.38	5.25 b	5.54 b	5.19 c	5.32
10 Gy	5.57 a	5.33 b	4.67 ab	5.19	6.63 a	5.96 b	5.75 b	6.11
20 Gy	4.35 c	6.17 a	5.02 a	5.18	4.36 c	7.63 a	6.35 a	6.11
30 Gy	3.23 d	3.36 cd	4.14 b	3.57	3.41 d	4.19 c	4.27 d	3.95
40 Gy	1.91 e	2.90 d	3.34 c	2.71	2.39 e	3.27 d	1.28 e	2.31
Means	4.032	4.222	4.376	–	4.408	5.318	4.568	–

Means followed by different letters in the same column indicate significant differences (p < 0.05).

Significant differences were observed in stomatal density of the genotypes. NIA-0819 showed the highest stomatal density of 53.56 mm^−2^ on the adaxial surface under 10 Gy while NIA-98 and BL4 recorded maximum stomatal densities (42.78 and 48.28 mm^−2^, respectively) under 20 Gy. Stomatal density for all the genotypes reduced under the maximum dose of gamma radiation. The mean values for adaxial stomatal density for NIA-0819, NIA-98, and BL4 were observed to be 46.05, 36.12, and 42.41 mm^−2^, respectively. The stomatal density was significantly higher at the abaxial surface as compared to the adaxial one. NIA-0819 showed the highest stomatal density of 115.31 mm^−2^ at the abaxial surface under 10 Gy, while BL4 and NIA-98 exhibited the maximum stomatal density of 95.92 and 88.82 mm^−2^ under 20 Gy, respectively (Table 3; Figs. 1, 2, 3).

**Table 3:** Mean values of the stomatal density (mm^-2^) on the adaxial and abaxial leaf surface of sugarcane genotypes.

Treatment	Stomatal density (mm^−2^)							
	Adaxial surface				Abaxial surface			
	NIA-0819	NIA-98	BL4	Mean	NIA-0819	NIA-98	BL4	Mean
Control	47.40 c	38.40 b	41.91 c	42.57	102.32 c	79.32 c	88.41 c	90.01
10 Gy	53.56 a	35.15 c	44.85 b	44.52	115.31 a	84.40 b	92.42 b	97.37
20 Gy	49.73 b	42.78 a	48.28 a	46.93	108.39 b	88.82 a	95.92 a	97.71
30 Gy	41.12 d	35.22 c	39.13 d	38.49	96.80 d	78.26 c	85.62 d	86.89
40 Gy	38.45 e	29.04 d	37.88 d	35.12	88.02 e	68.38 d	75.82 e	77.40
Means	46.053	36.121	42.412	–	102.17	79.839	87.643	–

Means followed by different letters in the same column indicate significant differences (p < 0.05).

The pooled agronomic performance of genotypes treated with gamma rays and the control are presented in Table 4. For any sugarcane breeding program targeting higher cane yield, improvement in major yield-contributing factors e.g. stalk weight, plant height, tillering potential, and stalk width is pivotal. In this study, the tallest stalks of 367.33 cm were observed in NIA-98 at 20 Gy, showing a significant increase over its control (324 cm). Similarly, stalks of 337.50 cm were harvested in NIA-0819 treated with 10 Gy. Minimum plant height (223 cm) was noted for NIA-98 at 40 Gy. Cane tillering and stool weight followed a similar trend as observed for the plant height. The highest number of stools per plant (8) were produced by 10 and 20 Gy treatments of NIA-0819. The genotypes having better tillering habit (in check) also showed a higher increment in lower doses of gamma rays. The maximum dose resulted in deterioration of the tillering potential of all the genotypes. Stool weight was observed to be maximum for 20 Gy treatments of NIA-98 (6.63 kg) and BL4 (8.35 kg); and 10 Gy dose of NIA-0819 (8.71 kg).

**Table 4:** Agronomic traits of sugarcane genotypes irradiated using four levels of gamma radiations.

Genotypes	Gamma radiation doses (Gy)	Plant height (cm)	Number of stools plant^-1^	Stool weight (kg)	Stool girth (cm)	Number of internodes	Internode length (cm)	Leaf length (cm)	Leaf width (cm)
NIA-0819	0	319.83 d	7.33 b	7.80 cd	3.06 b–e	22.16 b–d	17.00 bc	133.33 f	4.30 bc
	10	337.50 b	8.00 a	8.71 a	2.83 e–g	28.83 a	19.37 a	143.33 e	5.05 a
	20	323.00 c	8.00 a	8.43 ab	2.59 g	23.66 bc	17.70 bc	136.00 f	4.20 b–d
	30	317.00 d	6. 33 c	6.14 ef	2.83 e–g	24.50 b	14.33 d	132.83 f	4.17 b–d
	40	299.00 e	5.33 d	5.20 g	2.70 fg	20.66 de	12.23 f	123.83 h	3.77 e
NIA-98	0	324.00 c	5.00 d	4.99 g	2.68 fg	16.50 fg	17.66 bc	175.17 b	4.09 c–e
	10	290.83 e	6.33 c	4.99 g	2.92 d–f	17.16 fg	16.66 c	174.00 b	4.12 c–e
	20	367.33 a	6.00 c	6.63 d–f	3.01 c–e	21.00 c–e	18.33 ab	182.83 a	5.35 a
	30	247.83 g	4.66 e	4.76 g	2.92 d–f	16.66 fg	13.66 de	163.33 c	3.86 de
	40	223.00 i	3.66 f	3.69 h	2.71 fg	15.00 g	10.50 g	156.50 d	3.39 f
BL4	0	247.50 g	4.66 e	6.97 c–e	3.34 a	15.50 g	10.66 g	124.62 g	4.23 bc
	10	246.83 g	6.33 c	7.30 cd	3.25 a–c	15.50 g	10.50 g	119.35 g	4.26 bc
	20	259.50 f	7.00 b	8.35 ab	3.27 ab	18.50 ef	12.66 ef	123.35 gh	4.48 b
	30	245.17 h	5.33 d	6.06 f	3.24 a–c	15.50 g	9.50 g	119.06 hi	3.78 e
	40	237.50 h	3.33 f	4.76 g	3.10 a–d	14.83 g	7.83 h	114.70 i	3.35 f

Means followed by different letters in the same column indicate significant differences (p < 0.05).

The internode parameters also exhibited changes as a result of exposure to gamma radiations. Maximum internode length (19.37 cm), as well as the number of internodes (28.83), were recorded under 10 Gy treatment of NIA-0819. Moreover, leaf length and width of studied sugarcane genotypes also varied significantly. The leaves of NIA-98 at 20 Gy dose were 182.83 cm long on average, against 175.17 cm in control. NIA-98 produced 40–50 cm longer leaves than those of BL4 and NIA-0819; the width being almost the same. The maximum improvement in leaf width (5.35 cm) was seen in NIA-98 at 20 Gy (Table 4).

The juice quality parameters are also very important in sugarcane breeding. These traits determine the final sugar yield. Therefore, sugar yield, brix %, fiber %, sucrose %, commercial cane sugar % (CSS %), and purity % were analyzed (Table 5). The highest brix % values were observed for 30 Gy treatment of BL4 (19.61%). While the minimum brix (15.51%) was recorded for NIA-98 at 10 Gy. Moreover, the maximum fiber contents (15.10%) were noticed for NIA-0819 at 40 Gy. Whereas, the least fiber values were produced by NIA-98 (11.27%) under 30 Gy. Similarly, for CCS % and purity %, the maximum observations were recorded at higher doses. Furthermore, sucrose % also improved up to 14.54% in BL4 under 40 Gy as compared to 12.33% in control.

**Table 5:** Quality related traits of sugarcane genotypes irradiated with four levels of gamma radiations.

Genotypes	Gamma radiation doses (Gy)	Brix (%)	Fiber (%)	Sucrose (%)	Commercial cane sugar (%)	Purity (%)	Sugar yield (t ha^-1^)	Cane yield (t ha^-1^)
NIA-0819	0	16.83 d–g	12.72 bc	10.77 hi	6.54 i	64.31 d–f	4.80 e	77.96 cd
	10	16.25 g	13.34 b	11.01 h	5.52 j	69.27 b–d	4.60 ef	87.08 a
	20	18.18 b–d	14.80 a	11.63 fg	6.81 i	64.06 d–f	5.91 bc	84.33 ab
	30	16.60 fg	14.84 a	12.00 ef	8.35 g	72.76 a–c	4.90 e	61.43 ef
	40	17.78 c–f	15.10 a	12.20 d–f	8.71 f	68.64 cd	4.80 ef	52.00 g
NIA-98	0	16.02 g	11.72 c–e	10.35 ij	10.15 e	65.32 de	3.72 g	49.86 g
	10	15.51 g	12.25 b–e	11.28 h	11.64 c	73.55 a–c	5.08 dc	49.86 g
	20	16.78 e–g	11.46 de	9.85 j	8.72 f	58.79 fg	4.10 fg	66.30 d–f
	30	15.87 g	11.27 e	8.81 k	11.20 d	56.21 g	3.73 g	47.63 g
	40	16.56 fg	11.47 de	10.07 j	12.10 b	61.24 e–g	3.67 g	36.86 h
BL4	0	18.07 b–e	11.54 de	12.33 c–e	7.48 h	68.39 cd	5.91 c	69.73 c–e
	10	18.61 a–c	12.41 b–d	12.65 cd	7.44 h	67.97 cd	5.55 cd	72.96 cd
	20	18.08 b–e	12.24 b–e	12.86 c	8.14 g	65.59 de	6.75 a	83.46 ab
	30	19.61 a	11.91 c–e	13.62 b	10.28 e	75.54 a	6.52 ab	60.63 f
	40	19.32 ab	11.81 c–e	14.54 a	13.16 a	75.28 ab	6.14 a–c	47.63 g

Means followed by different letters in the same column indicate significant differences (p < 0.05).

Cane yield and sugar yield determine the economic returns of the sugarcane crop. Cane yield is the result of agronomic parameters; whereas, regarding sugar yield, the agronomic as well as quality traits come into play. BL4 showed the maximum sugar and cane yield of 6.75 and 83.46 t ha^−1^ under 20 Gy of gamma radiation. For NIA-98, the highest sugar (5.08 t ha^−1^) and cane yield (66.30 t ha^−1^) were observed at 10 and 20 Gy, respectively. Whereas, NIA-0819 produced the maximum sugar yield of 5.91 t ha^−1^ (at 20 Gy) and cane yield of 87.08 t ha^−1^ (under 10 Gy). The 40 Gy treatment produced severe lethal effects in NIA-98 resulting in the lowest sugar and cane yield of 3.67, and 36.86 t ha^−1^, respectively (Table 5).

Percent change in the stomatal parameters, as well as sugar and cane yield, were also assessed (Table 6). For adaxial and abaxial stomatal lengths, the maximum percent rise for NIA-98 and BL4 was observed under 20 Gy; whereas, NIA-0819 showed the highest percent increase at 10 Gy. Since NIA-98 and BL4 are late and mid maturing varieties (respectively) vs. NIA-0819 (an early maturing variety), such variations depicted a possible role of genetic factors towards percent changes in stomatal characteristics. For abaxial stomatal length, the maximum percent increase of 42.86% was observed in NIA-0819, while for the adaxial surface, the same genotype exhibited a maximum percent rise of 49.97%. A significant percent decrease in stomatal length was observed at 30 and 40 Gy in all the genotypes. Similar trend was observed for stomatal widths as well. Regarding adaxial stomatal density, the maximum rise was observed in BL4 (15.19%) at 20 Gy, followed by NIA-0819 at 10 Gy (12.99%). The maximum decline of 24.37% was demonstrated by NIA-98 at 40 Gy. For abaxial surface, the maximum increase in stomatal density was recorded in NIA-0819 (12.69%) at 10 Gy. The highest percent increase in cane yield for NIA-98 (32.97%) and BL4 (19.69%) was attained at 20 Gy, while NIA-0819 showed maximum rise at 10 Gy (11.69%). Under 40 Gy, the cane yield deteriorated by 33.30%, 26.07%, and 31.69% for NIA-0819, NIA-98, and BL4, respectively. Regarding sugar yield, NIA-0819, and BL4 recorded the maximum percent surge of 23.13 and 14.21% (at 20 Gy), respectively, whilst NIA-98 showed the maximum percent increment of 36.55% (at 10 Gy). Percent rise in sugar yield was observed in all radiation treatments except 10 Gy of NIA-0819 and 40 Gy of NIA-98. The results indicated that an increase in sugar contents related parameters of the genotypes at higher doses of gamma radiation contributed to maintaining the sugar yield despite the reduction in cane yield (Table 7).

**Table 6:** Percent change in stomatal length, width, and density of sugarcane genotypes irradiated with four levels of gamma radiations.

Treatment ( Gy)	Adaxial stomatal length			Abaxial stomatal length			Adaxial stomatal width			Abaxial stomatal width		
	NIA-0819 (%)	NIA-98 (%)	BL4 (%)	NIA-0819 (%)	NIA-98 (%)	BL4 (%)	NIA-0819 (%)	NIA-98 (%)	BL4 (%)	NIA-0819 (%)	NIA-98 (%)	BL4 (%)
10	49.97	11.61	13.85	42.86	4.66	8.57	9.21	− 0.59	− 0.84	26.28	7.58	10.78
20	29.23	41.20	35.47	25.66	27.63	26.62	− 14.70	84.17	6.58	− 16.95	37.72	22.35
30	− 6.26	− 12.55	− 14.49	− 18.10	− 12.18	− 4.48	− 33.13	0.29	− 29.10	− 38.47	− 24.36	− 17.72
40	− 27.63	− 38.33	− 33.60	− 35.99	− 39.49	− 36.04	− 62.54	− 13.43	− 12.08	− 54.47	− 40.97	− 75.33

**Table 7:** Percent change in stomatal density, cane yield, and sugar yield of sugarcane genotypes irradiated with four levels of gamma radiations.

Treatment (Gy)	Adaxial stomatal density			Abaxial stomatal density			Cane yield			Sugar yield		
	NIA-0819 (%)	NIA-98 (%)	BL4 (%)	NIA-0819 (%)	NIA-98 (%)	BL4 (%)	NIA-0819 (%)	NIA-98 (%)	BL4 (%)	NIA-0819 (%)	NIA-98 (%)	BL4 (%)
10	12.99	− 8.46	7.01	12.69	6.40	4.53	11.69	0	4.63	− 4.17	36.55	− 6.09
20	4.90	11.40	15.19	5.93	11.97	8.49	8.17	32.97	19.69	23.13	10.22	14.21
30	− 13.24	− 8.28	− 6.63	− 5.39	− 1.33	− 3.15	− 21.20	− 4.47	− 13.05	2.08	0.27	10.32
40	− 18.88	− 24.37	− 9.61	− 13.97	− 13.79	− 14.24	− 33.30	− 26.07	− 31.69	0	− 1.34	3.89

**Figure 4:**
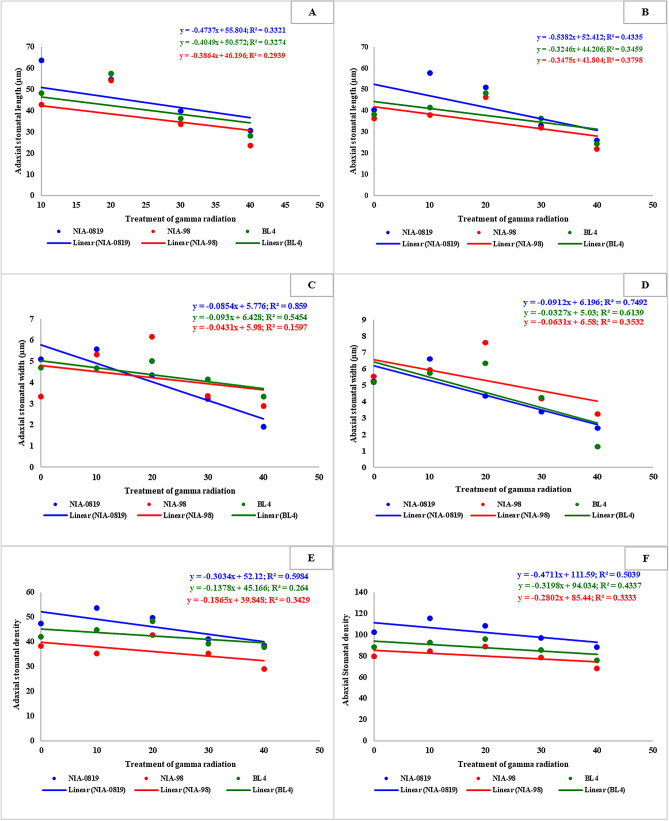
Coefficient of determination for stomatal parameters i.e. adaxial stomatal length (**A**), abaxial stomatal length (**B**), adaxial stomatal width (**C**), abaxial stomatal width (**D**), adaxial stomatal density (**E**) and abaxial stomatal density (**F**).Regression analysis of stomatal parameters against the doses of gamma radiation was conducted using Microsoft Excel v. 2019. The regression slope for stomatal length, width, and density on both surfaces of sugarcane leaf indicated a negative trend. Nevertheless, the degree of change was highly dependent on genotypes under the exposure of gamma radiations.

**Figure 5:**
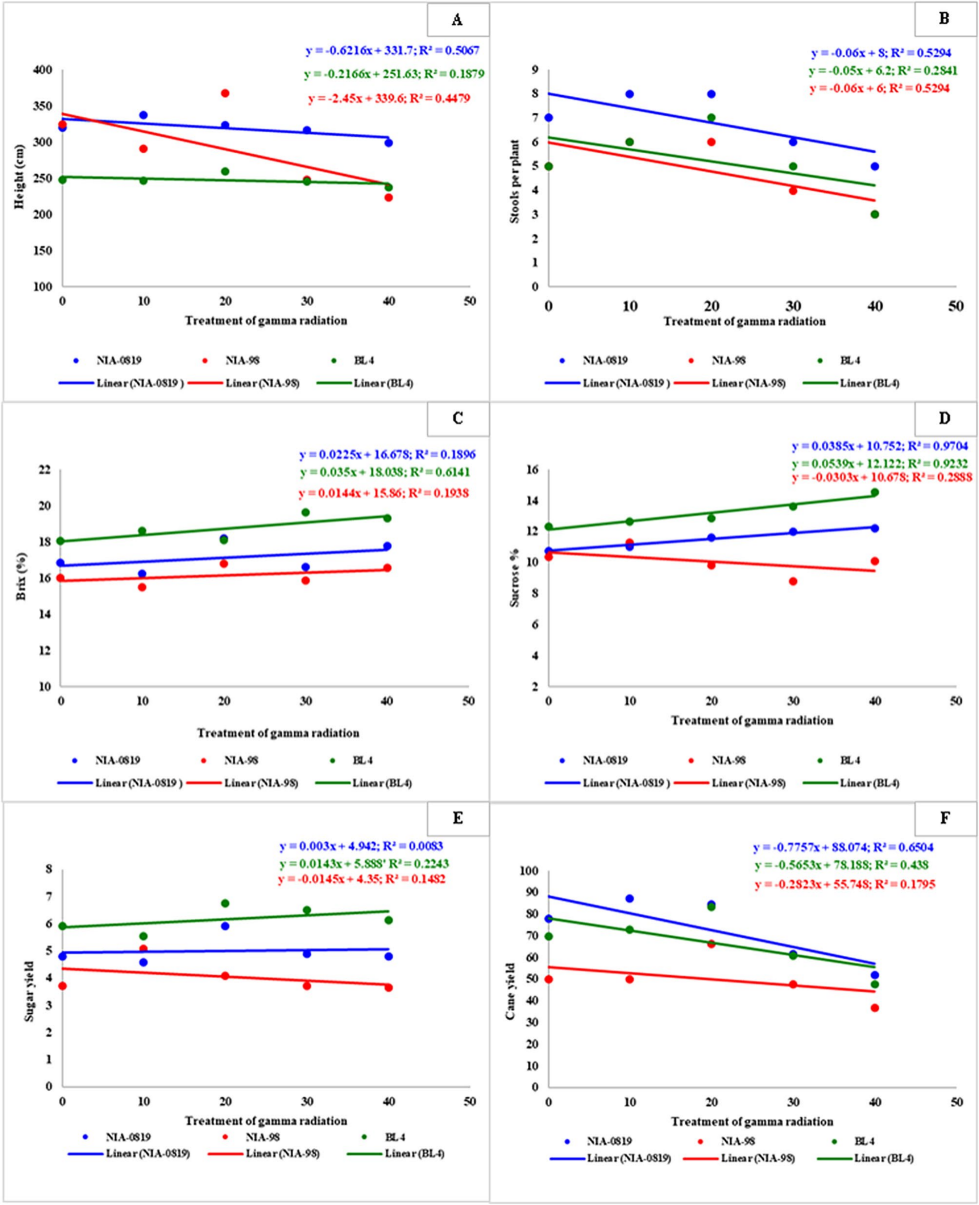
Coefficient of determination for sugarcane height (**A**), stools per plant (**B**), Brix % (**C**), Sucrose % (**D**), sugar yield (**E**) and cane yield (**F**). Regression analysis of stomatal density against the doses of gamma radiation was conducted using Microsoft Office v. 2019. A similar trend was observed for cane yield of different genotypes; however, the degree of change varied. Sugar yield, on the other hand, showed an upwards trend for NIA-0819 and BL4 against a downwards trend for NIA-98.

The major aim of this study i.e. investigation of the association of physical mutagenesis with the stomatal parameters, and the influence of these traits on crop performance, was dissected through regression and correlation analysis. Regression plots indicated that the lower doses of gamma radiations (10 and 20 Gy) had a stimulating effect on certain traits while the crop characteristics declined under higher doses (30 and 40 Gy). However, in general, the regression equations represented a negative slope between the stomatal traits vs. the doses of gamma radiation (Fig. 4). Hence, the intercept values for stomatal length, width, and density on both surfaces of the sugarcane leaf were negative. Maximum R^2^ among stomatal traits was observed for the adaxial stomatal width of NIA-0819 (0.859) while the least value for the coefficient of regression was shown by NIA-98 for the same parameter (0.1597). Nevertheless, the degree and pace of declining slope were highly dependent on genotypes exposed to gamma radiations. A similar trend was observed for agronomic traits as well (Figs. 5 A and B; Supplementary Figs. S1–S8). Yet, contribution by the genetic factors against response to physical mutagenesis was more evident for these characteristics. For instance, the intercept for height was highly negative for NIA-98 (− 2.45) as compared to the other two genotypes which showed a minute decline. The quality-related characteristics of sugarcane showed relatively different trend as the intercepts for many of such traits were positive (Fig. 5C,D, Supplementary Figs. S9–S13). The intercept was positive for all three varieties regarding brix % while for sucrose %, only NIA-98 showed a negative slope. Such behavior of genotypes plays a paramount role in overall sugar yields, which was evident from the regression analysis of sugar and cane yields (Fig. 5E,F). The cane yield showed a negative slope—as observed for most of the yield-contributing parameters. However, the intercept for sugar yield was weakly positive for NIA-0819 (R^2^ = 0.0083; intercept = 0.003) and BL4 (R^2^ = 0.0143; intercept = 0.2243) indicating a significant role of a rising slope of sugar-quality related traits towards sugar yield (Fig. 5E,F).

Correlation analysis of physical mutagenesis with the stomatal and agronomic parameters gave interesting insights into the data (Table 8). Even though the initial two doses of gamma radiation had shown an increase in agronomic parameters, generally, the overall correlation seemed to be negative. Contrarily, the juice quality traits exhibited weak positive correlation vs. the doses of gamma radiation. The correlation coefficient of stomatal traits with the agronomic data and cane yield was positive. Nevertheless, these traits generally showed negative correlation with the sugar parameters. Quantitative traits viz., height, girth, tillers, and internode divulged positive association among each other, while qualitative parameters (brix %, sucrose %, CCS %, and purity %) also illustrated positive correlation with each other. However, both of these two classes of characteristics correlated negatively. The cane yield was seen to highly correlate with stool weight, tillering habit, and leaf width (1, 0.8865, and 0.6818, respectively). On the other hand, sugar yield had positive correlation with sugar parameters but its correlation with stomatal traits was either very weak, or negative. Sucrose % and brix % were observed to be the most import traits towards sugar yield (0.8673, and 0.7993, respectively).

**Table 8:** Correlation analysis of doses of gamma radiation, stomatal parameters, yield attributes and sugar-quality traits.

	Gamma radiation doses (Gy)	Stomatal length (Adaxial)	Stomatal length (Abaxial)	Width of stomatal aperture (Adaxial)	Width of stomatal aperture (Abaxial)	Stomatal density (Adaxial)	Stomatal density (Abaxial)	Plant height	Number of stools plant^-1^	Stool girth	Number of internodes	Internode length	Leaf length	Leaf width	Stool weight	Brix	Fiber	Sucrose	Commercial cane sugar	Purity	Sugar yield	Cane yield
Gamma Radiation Doses (Gy)	**1**																					
Stomatal Length (Adaxial)	− 0.5386*	1																				
Stomatal Length (Abaxial)	− 0.5891*	0.9793	1																			
Width of stomatal aperture (Adaxial)	− 0.6498**	0.7464**	0.7378**	1																		
Width of stomatal aperture (Abaxial)	− 0.656**	0.7073**	0.7262**	0.8667**	1																	
Stomatal density (Adaxial	− 0.476	0.871**	0.872**	0.431	0.4295	1																
Stomatal density (Abaxial)	− 0.4173	0.819**	0.871**	0.3671	0.348	0.9326	1															
Plant height	− 0.3618	0.558*	0.5532*	0.3836	0.5752*	0.5005*	0.5841*	1														
Number of Stools Plant^-1^	− 0.5354*	0.8918**	0.8979**	0.5781*	0.5102	0.8765**	0.9366**	0.6464	1													
Stool Girth	− 0.2322	0.1379	0.1092	0.3336	0.1505*	0.1575	− 0.0348	− 0.442	− 0.0534	1												
Number of Internodes	− 0.1403	0.6133*	0.6069*	0.1722	0.2804	0.6864**	0.842	0.7642*	0.7523**	− 0.4144	1											
Internode Length	− 0.5111*	0.6121	0.6469**	0.5257*	0.6861**	0.4586	0.5626*	0.8852**	0.6873**	− 0.4974	0.7059	1										
Leaf Length	− 0.207*	0.0544**	0.0758	0.4191	0.5711*	− 0.3135	− 0.2494	0.4444	− 0.0375	− 0.4507*	0.039	0.6295*	1									
Leaf Width	− 0.5339*	0.8711**	0.8361**	0.8201**	0.8383**	0.6844	0.6489	0.7268	0.7282**	0.1129	0.6054**	0.7024	0.3229	1								
Stool Weight	− 0.4988*	0.8826*	0.8825**	0.5266*	0.4251	0.9696**	0.9119	0.4037	0.8865**	0.2641	0.6048*	0.3892*	− 0.3406	0.6809*	1							
Brix	0.2747	− 0.0678	− 0.0728	− 0.2834	− 0.3839	0.1796	0.0137	− 0.435	− 0.1105	0.5135*	− 0.3068	− 0.6761**	− 0.8*	− 0.2826	0.2242	1						
Fiber	0.1785	0.1918	0.1517	− 0.3135	− 0.3589	0.389*	0.593*	0.4203	0.4949*	− 0.4876*	0.6769**	0.2469	− 0.3396	0.05	0.3359	0.0546	1					
Sucrose	0.1993	− 0.0479	− 0.0927	− 0.3433	− 0.3817	0.1711	0.0685	− 0.3743	− 0.044	0.4849	− 0.2044	− 0.6153*	− 0.8162**	− 0.3016	0.1839	0.8379**	0.1936	1				
Commercial Cane Sugar	0.5395*	− 0.7511**	− 0.7535**	− 0.4449*	− 0.3289	0.8714**	0.8964**	− 0.5194*	− 0.841**	− 0.0642	− 0.6972**	− 0.4758*	0.2483	− 0.6782*	− 0.8872**	− 0.0215	− 0.4794*	0.0399	1			
Purity	0.0677	− 0.0992	− 0.1126	− 0.3296	− 0.2521	0.0322	0.0846	− 0.1709	− 0.0043	0.2529	− 0.0397	− 0.3488	− 0.5244*	− 0.267	0.0119	0.4369	0.2606	0.8084	0.1092	1		
Sugar yield	0.0134	0.2448	0.2235	− 0.0146	− 0.3817	0.3834	0.2755	− 0.3286	0.2347	0.6024	− 0.1287	− 0.4445*	− 0.7562**	− 0.088	0.4656*	0.7993**	0.1398	0.8673**	− 0.1577	0.5947*	1	
Cane yield	− 0.5037	0.8824**	0.8847**	0.5298*	0.4279	0.9705**	0.9124**	0.4029	0.8865	0.2636*	0.6055	0.3961	− 0.3333	0.6818**	0.9995**	0.2126	0.3286	0.1664	− 0.8883	− 0.0061	0.4546	1

## Discussion

Selection for yield parameters is an arduous job because of large inter-plot differences and error variance^40^. For precise selection, support of agronomic attributes along with the endomorphic characteristics can ease the breeding chore. Among the endomorphic traits, stomatal parameters can be excellently employed for the purpose as a balanced gaseous exchange for photosynthetic activity and controlled water losses from transpiration are major components of crop productivity, especially in stress conditions^41–44^. This study investigated the effects of different doses of gamma radiation on stomata of sugarcane and explored their association with the yield and yield-contributing parameters. As per our knowledge, this is the first report investigating the direct impact of mutagenesis on sugarcane stomata through SEM and resulting changes in crop performance; and hence, carries paramount importance for sugarcane breeding and stress tolerance research.

Using four mutagenic treatments, viz. 10, 20, 30, and 40 Gy of gamma rays, it was observed that the stomatal size increased at lower doses; whereas, it reduced at higher doses. Similar observations were recorded for stomatal density as well. Such diminution and augmentation in stomatal parameters could be ascribed to the stimulating effect at 10 and 20 Gy of gamma rays; and higher frequency of the damage under 30 and 40 Gy^45^. The decline in the number of stomata under elevated doses of mutagen has earlier been described by Qosim et al.^46^ as well. A similar trend was noted for the cane yield and yield-contributing characters. Hence, the results reflected higher opportunities for the selection of desirable mutants at lower doses^32^. In agreement with our report, lower doses of gamma radiations have been reported to produce desired mutations in other crops too^47–49^. On the other hand, sugar-related parameters like sucrose %, CCS %, and purity % were observed to increase even at higher doses, indicating a positive impact on these traits. Such behavior of sugar-related characteristics also resulted in an interesting response towards the ultimate sugar yield^32^. For instance, the sugar yield of BL4 seemed to increase by 3.89 units at the highest dose (40 Gy). Many of the intercept values were also observed to be positive. At this level of gamma radiation, the yield and yield-contributing traits had significantly declined. These results hinted that a reduction in vegetative growth can enhance the quality-related characters of sugarcane. Similar results were earlier reported by Khan et al.^32,50^. Kottapalli^51^ also accounted that elevated amount of sucrose induces bulging of guard cells which leads to the closure of stomata. Such change ultimately reduces gaseous exchange causing perturbed photosynthetic activity and instigating low sink production. It can also be presumed that the low cane yield of high sugar content genotypes maybe because of the decimal behavior of stomata caused by high sucrose content^52^.

The role of genotypic makeup against response to doses of radiation was evident from the varying response of genotypes. While assessing the agronomic traits, NIA-0819 exhibited positive effects at 10 Gy only, while the other two varieties, BL4 and NIA-98 showed augmentation in various traits till 20 Gy of gamma radiation. The variable response was also apparent from different regression slopes whilst analyzing the effect of the same level of doses. The disparity in outcomes of mutagenic activity on these genotypes reflected the different genetic buffering capacity of the varieties^53^. On correlation analysis, the stomatal characteristics showed a positive association with the agronomic parameters. Contrarily, the juice quality traits exhibited weak positive correlation vs. the doses of gamma radiation. The correlation of agronomic traits with the quality parameters was generally negative which has been earlier reported by Khan et al.^54^ and Seema et al.^55^. The cane yield seemed to be highly dependent on stool weight, tillering habit, and height which agreed to the report of Soomro et al.^56^. Sugar recovery was observed to be negatively correlated with quantitative traits parallel to the study of Khan et al.^57^. The dependency of sugar yield on sucrose and brix % has been described by Khan et al.^58^ as well.

Various studies have demonstrated the role of stomata in biomass accumulation and crop yield determination. Sarwar et al.^24^ reported a significant and positive correlation of stomatal length, width, and density with the grain yield in rice. Parallel to our study, they demonstrated positive regression plots between yield and stomatal traits. Our results revealed an increase in stomatal density and aperture size under low doses of gamma rays. Moreover, improvement in the agronomic performance of the genotypes was also observed under the same doses. This was in agreement with several researchers who have proposed that such enhancement in the stomatal traits facilitates gaseous exchange, high photosynthetic activity, and additional sink formation that ultimately results in higher crop yields^24,59^. The cane yield showed a significant positive correlation with the number, length, and width of the stomatal aperture. Profuse documentation on correlations between stomatal features and yield are available in other crops^59–62^. Buckley^63^ reported that the photosynthetic activity of the plant mainly depends on the number of stomata in the leaf. Chandra and Das^42^ observed that the length and width of the stomata in rice significantly affect the photosynthesis. Moreover, Maherali et al.^64^ reported that larger stomata with wide aperture facilitate more carbon dioxide intake in the plant, resulting in increased carbon diffusion and enhanced sink. Qosim et al.^46^ also reported that the enlarged stomatal length and width in plants improve photosynthetic activity. Furthermore, Limochi and Eskandari^59^ mentioned that stomatal count and its size directly relate to the crop output. Hence, the stomatal characters can be used in breeding programs for enhancing crop yields; as irradiation treatments cause alterations in stomata and may be exploited as an approach for creating superior mutants.

Physical mutagenesis has been extensively employed for sugarcane improvement and has resulted in the successful release of various commercial varieties^32^. However, there is hardly any information on mutagenic effects with reference to endomorphic traits^41^. Chandra and Das^42^ illustrated that stomatal size in rice influences the rate of photosynthetic activity, as the larger stomata exhibit more gaseous exchange as compared to the smaller ones. They also reported that larger stomata are mostly present in the broad leaves as compared to narrow leaves; the broadleaf cultivars have been proposed to be high yielding by Ohsumi et al.^43^. Moreover, Faralli et al.^44^ stated that changes in stomatal characteristics impact two major crop yield determinants of wheat i.e. the cumulative rate of photosynthesis and water use efficiency. Regarding sugarcane, this is one of the first reports investigating the impact of gamma rays-induced mutagenesis on stomatal traits. The changes in the stomatal number, size, and density due to mutagenic effects also alter the plant’s morphological response, apart from its yield behavior; as stomata play a vital role in stress response as well^28^. The increased stomatal resistance assists the plant to conserve water under drought conditions^65,66^. The environmental factors such as light, temperature, and CO_2_ concentration also influence the stomatal response to water stress. Lawson and Vialet-Chabrand^67^, and Vialet-Chabrand and Lawson^68^ documented that the higher surface area/volume ratio provides faster ion fluxes, which would facilitate the faster variations in the turgor of guard cells causing a rapid stomatal reaction. However, Franks and Beerling^2^ and Lawson and Blatt^69^ proposed that smaller stomatal size offers a rapid aperture response. Nevertheless, the role of stomatal parameters against stress response needs to be dissected through future studies.

With the rapid rise in the global population, the demand for high yielding as well as stress-tolerant crop varieties is increasing. Moreover, sugarcane has recently emerged as a biorefinery, and its role in bioenergy production has also been recognized^70,71^. Genetic improvement in sugarcane mainly involves asexual breeding methods^72^. Cane and sugar yield has been static for many years in most of the cane growing countries because of germplasm exhaustion. To develop new and improved sugarcane varieties, novel avenues of germplasm development and selection need to be adopted^36^. Besides, water availability has already become a limiting factor in crop production. Water stress-tolerant and high yielding genotypes are the demand of the day in changing climate scenario.

## Conclusion

The results of this study indicated that the gamma rays irradiation treatments cause alterations in stomata. Such changes in stomatal traits can be exploited as an approach for creating superior mutants for sugarcane improvement. The gamma rays triggered variations in stomatal density, length, and width. The stomatal size increased at lower doses; whereas, it reduced at higher doses. Stomatal density also showed a similar trend. Hence, it was inferred that the lower doses of gamma radiation produced a stimulating effect. The changes in stomatal parameters correlated with the agronomic performance of the sugarcane genotypes. The cane yield showed a significant positive correlation with the number, length, and width of the stomata. Hence, it was concluded that stomatal aspects of sugarcane crop could play an important role in selection. Earlier, Shahinnia et al.^73^ employed QTL mapping for stomatal traits and reported overlaps between stomatal QTLs and yield across environments. Such support form stomatal traits for selection can markedly improve the pace of sugarcane breeding and may open new avenues for sugarcane improvement through novel emerging techniques such as CRISPR-Cas mediated mutagenesis and genome editing. Moreover, these investigations may help in understanding the role of stomata towards sugarcane productivity.

## Supplementary information

Supplementary Information 1
