# Supplementary material for: Revisiting the physical mutagenesis for sugarcane improvement: a stomatal prospective
Source: Sci Rep. 2020 Sep 29;10:16003. doi: 10.1038/s41598-020-73087-z (PMC7524725; doi:10.1038/s41598-020-73087-z)

***Supplementary Material***

**Revisiting the Physical Mutagenesis for Sugarcane Improvement: A Stomatal Prospective**

**(Research Article)**

**SCIENTIFIC REPORTS**

**Shafquat Yasmeen, Muhammad Tahir Khan and Imtiaz Ahmed Khan***

Sugarcane Biotechnology Group, Nuclear Institute of Agriculture (NIA), Tandojam, 70060, Pakistan

*****Corresponding author**

**Dr. Imtiaz Ahmed Khan**

**Email:** [imtiazkhan2@gmail.com](mailto:imtiazkhan2@gmail.com)

Supplementary Material


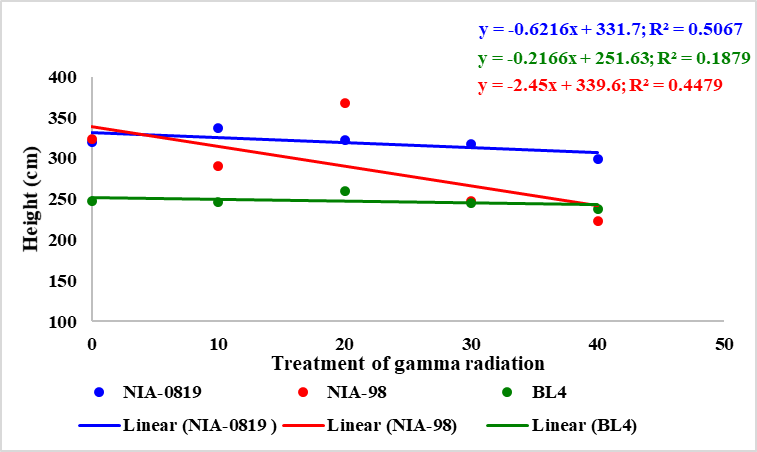


**Fig. S1** Coefficient of determination for sugarcane height. Regression analysis of sugarcane plant height against the doses of gamma radiation was conducted using Microsoft Excel v. 2019. The slope was seen to be negative for all the varieties. R^2^ value was lowest for BL4 and highest for NIA-0819. Nevertheless, the trend line varied among genotypes depicting only a slight decline for BL4.


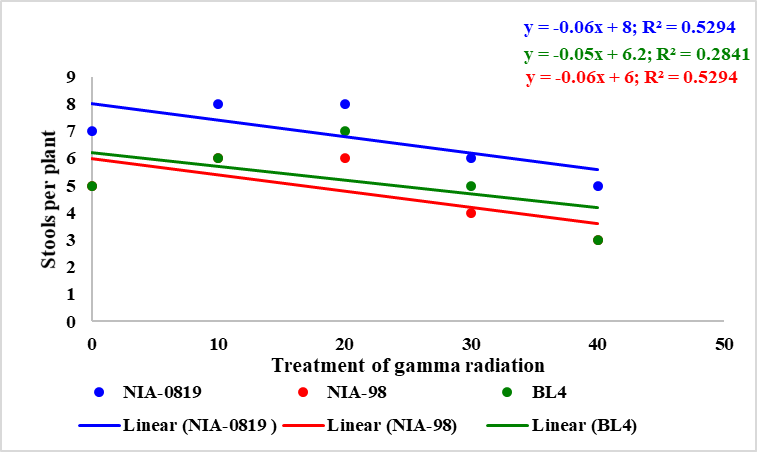


**Fig. S2** Coefficient of determination for sugarcane tillering. Regression analysis of sugarcane tillering against the doses of gamma radiation was conducted using Microsoft Excel v. 2019. The slope for all the genotypes exhibited a negative trend. R^2^ value was seen to be lowest for BL4. While coefficient of determination for NIA-98 and NIA-0819 was equal.


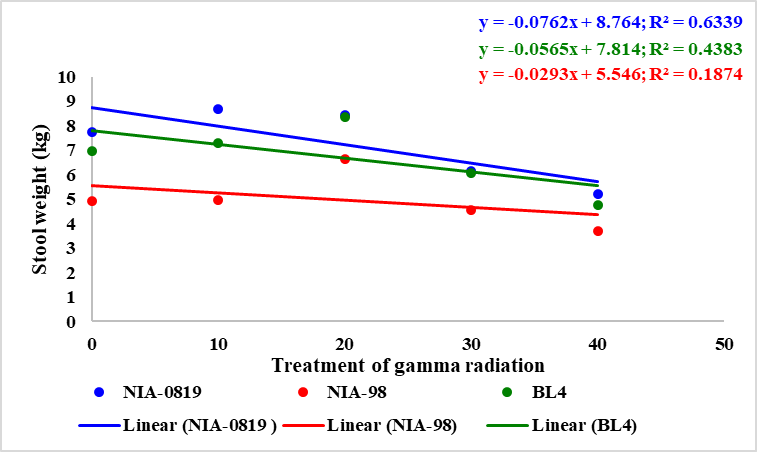


**Fig. S3** Coefficient of determination for plant weight. Regression analysis of sugarcane plant weight against the doses of gamma radiation was conducted using Microsoft Excel v. 2019. Slope for all the genotypes performed negatively. However, the slopes depicted varietal variations as well against the doses of gamma radiation.


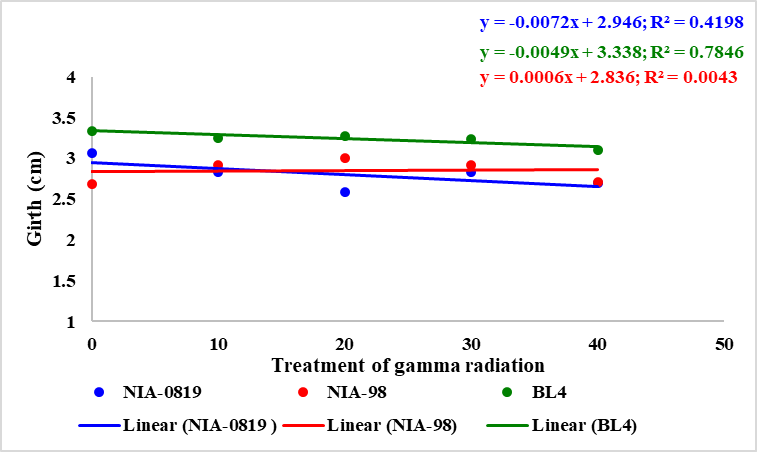


**Fig. S4** Coefficient of determination for sugarcane girth. Regression analysis of sugarcane girth against the doses of gamma radiation was conducted using Microsoft Excel v. 2019. The coefficient of determination was observed to be extremely low for NIA-98, while highest value was shown by BL4.


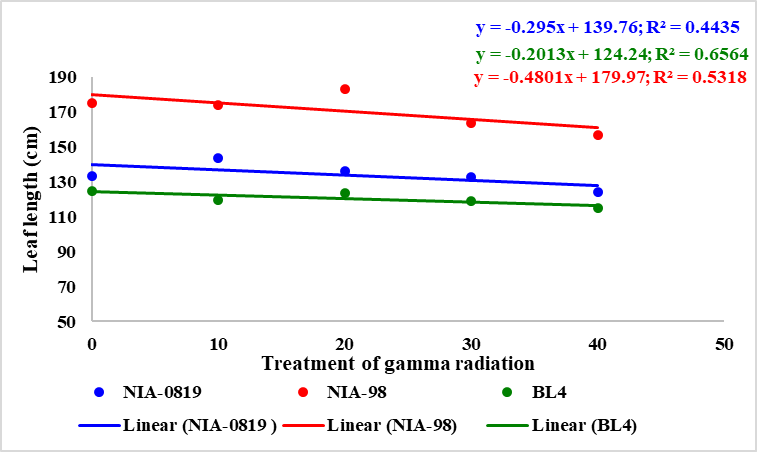


**Fig. S5** Coefficient of determination for leaf length. Regression analysis of sugarcane leaf length against the doses of gamma radiation was conducted using Microsoft Excel v. 2019. The slopes indicated a negative trend. Nevertheless, coefficient of determination was seen to be highest for BL4.


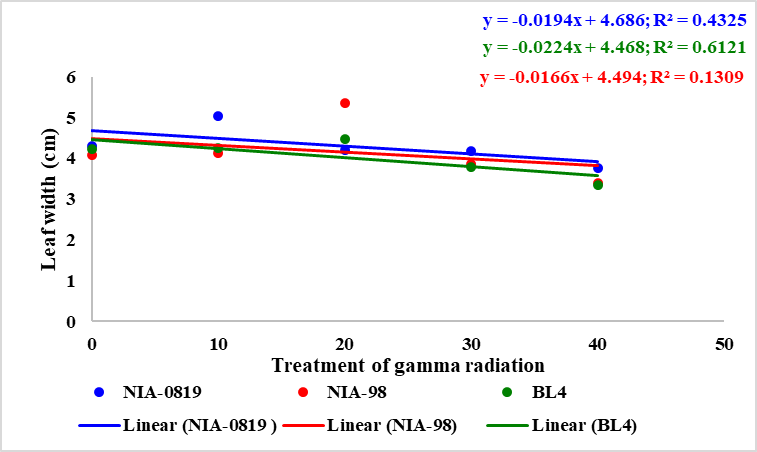


**Fig. S6** Coefficient of determination for leaf width of sugarcane. Regression analysis of sugarcane leaf width against the doses of gamma radiation was conducted using Microsoft Excel v. 2019. Slopes for all the genotypes showed a negative trend.


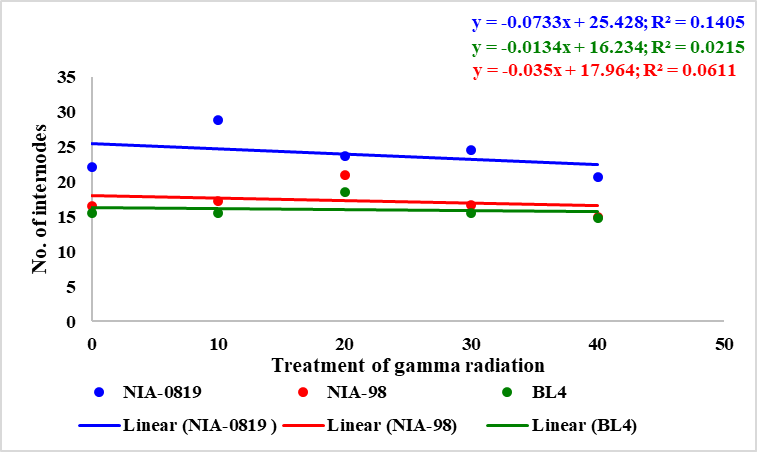


**Fig. S7** Coefficient of determination for number of internodes. Regression analysis for number of internodes of sugarcane against the doses of gamma radiation was conducted using Microsoft Excel v. 2019. The R^2^ value was lowest for BL4. The regression equation for all the genotypes was negative.


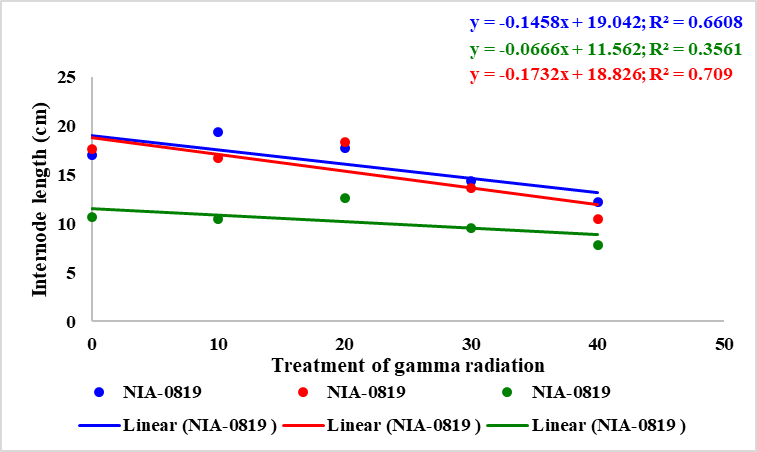


**Fig. S8** Coefficient of determination for internode length. Regression analysis of internode length of sugarcane against the doses of gamma radiation was conducted using Microsoft Excel v. 2019. All three regression equations were seen to be negative. The coefficient of determination was highest for NIA-0819.


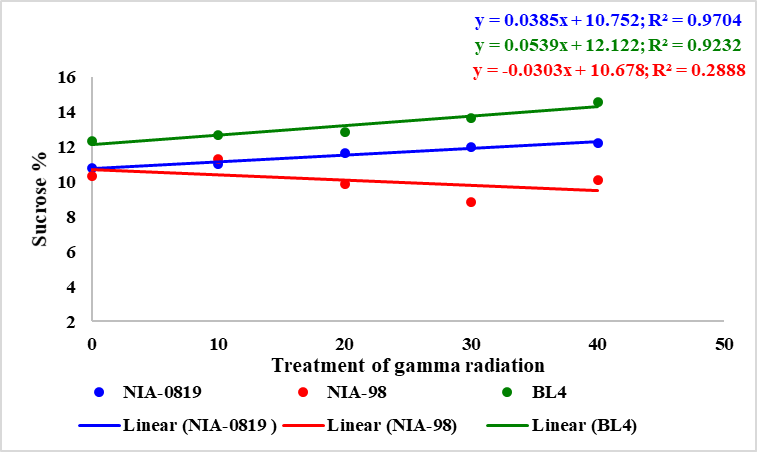


**Fig. S9** Coefficient of determination for sucrose % of sugarcane. Regression analysis of sugarcane sucrose % against the doses of gamma radiation was conducted using Microsoft Excel v. 2019. The regression equation was seen to be negative for NIA-98 while NIA-0819 and BL4 exhibited a positive regression equation.


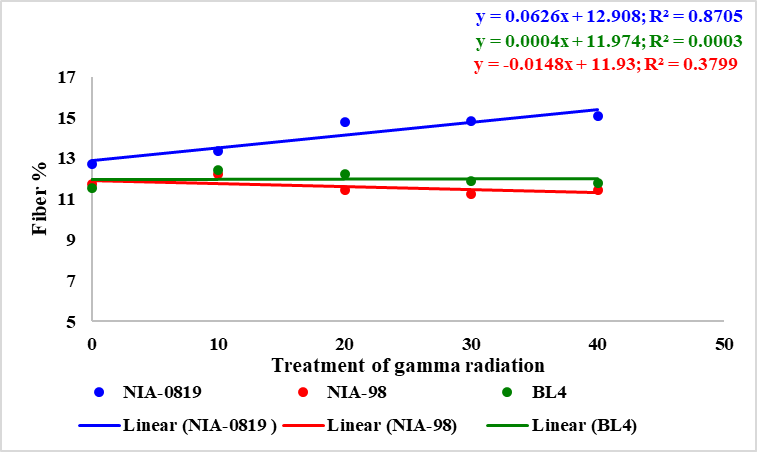


**Fig. S10** Coefficient of determination for fiber % of sugarcane. Regression analysis of sugarcane fiber % against the doses of gamma radiation was conducted using Microsoft Excel v. 2019. The regression equation was seen to be negative for NIA-98 while NIA-0819 and BL4 exhibited a positive regression equation. However, the coefficient of determination was extremely low for BL4.

**Fig. S11** Coefficient of determination for brix % of sugarcane. Regression analysis of sugarcane brix % against the doses of gamma radiation was conducted using Microsoft Excel v. 2019. For this parameter, all the genotypes under study exhibited a positive slope. The coefficient of determination was seen to be highest for BL4.


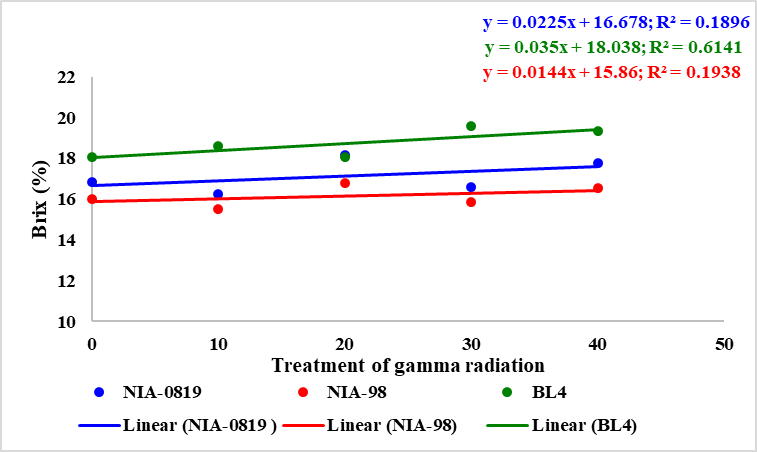


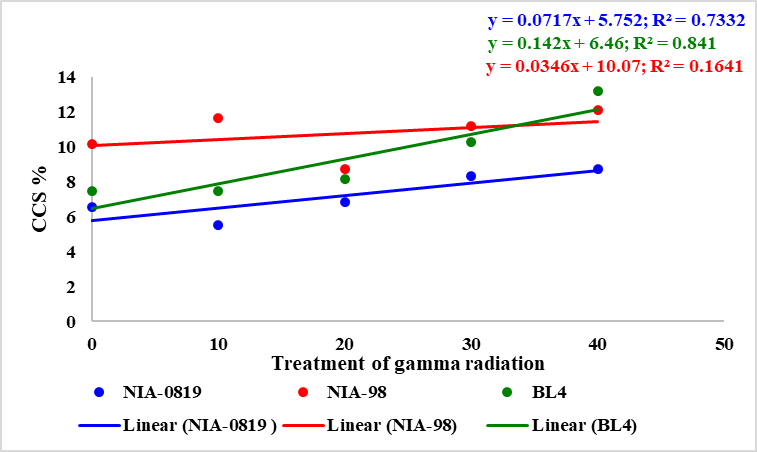


**Fig. S12** Coefficient of determination for commercial cane sugar (CCS) %. Regression analysis of sugarcane CCS % against the doses of gamma radiation was conducted using Microsoft Excel v. 2019. All three genotypes under study exhibited a positive slope. However, the highest coefficient of determination was shown by BL4.

**Fig. S13** Coefficient of determination for purity %. Regression analysis of sugarcane purity % against the doses of gamma radiation was conducted using Microsoft Excel v. 2019. An interesting variation was observed among the genotypes for this trait against the gamma radiation. NIA-98 showed a negative slope while the other two genotypes showed a positive trend.


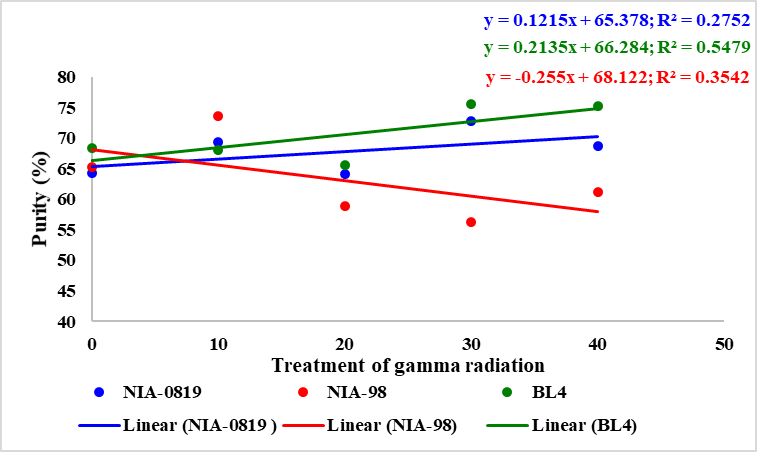

Supplement: Supplementary file 1 — Supplementary Information 1 [file 41598_2020_73087_MOESM1_ESM.doc]
